# Raman Spectroscopy of Changes in the Tissues of Teeth with Periodontitis

**DOI:** 10.3390/diagnostics10110876

**Published:** 2020-10-28

**Authors:** Elena Timchenko, Pavel Timchenko, Larisa Volova, Oleg Frolov, Maksim Zibin, Irina Bazhutova

**Affiliations:** 1 Department of Laser and Biotechnical Systems, Samara National Research University, 443086 Samara, Russia; Timpavel@mail.ru; 2 Research and Production Center “Samara Tissue Bank”, Samara State Medical University, 443079 Samara, Russia; volovalt@yandex.ru; 3 Department of Physics, Samara National Research University, 443086 Samara, Russia; owl-63@ya.ru; 4 «DIAMANT» Dental Clinic, 443090 Samara, Russia; zybin_m.a@mail.ru; 5 Department of Dentistry, Samara State Medical University, 443079 Samara, Russia; docba@mail.ru

**Keywords:** raman spectroscopy, optical diagnostic, periodontitis, tooth tissues, biophotonics, calculus

## Abstract

The results of experimental studies of the tissues of teeth with periodontitis, using the Raman spectroscopy method, are presented in this work. Spectral changes in the tissues of teeth with periodontitis were identified, and the results can be used for the correction of treatment of this disease in dental practice. Criteria for the noninvasive diagnosis of periodontitis, based on changes in tooth enamel spectral properties, were developed.

## 1. Introduction

Chronic periodontitis is a serious and widespread periodontal pathology that causes significant impairment to dentoalveolar system functions, with damage to supporting tooth structures and the loss of teeth [1]. Periodontitis is mostly spread (60–65%) among people over age 30 [2]. However, the percentage of young patients with a severe form of chronic periodontitis has increased to 11.2%, and among people over age 65, it is 30% [3]. Current data indicate that periodontitis is a polyethiological disease [4]. Periodontitis is an insidious disease because the initial signs of inflammatory processes often remain unnoticed, and the chronic condition causes serious consequences not only for the dentoalveolar system, but for the patient as a whole.

Prompt diagnosis and prevention through the treatment of patients with periodontal diseases is essential. To improve this process and allow for a noninvasive diagnosis of periodontitis, it is necessary to identify the structural changes that occur in the tissues of teeth with this disease. Most studies that have investigated changes caused by this disease have not focused on changes in hard dental tissues, but have investigated the surrounding soft tissues [5,6], oral fluid [6,7,8], or osseous tissue regeneration during periodontitis treatment [9]. Few studies have focused on the tissues of teeth with periodontitis. The authors of [10] showed that the main structural change in the tissues of teeth with generalized periodontitis is dentin mineralization, and the process of root canal treatment of such teeth is recommended as an additional means of avoiding progression to dentin demineralization. The authors of [11] noted an increase in the micro-hardness of enamel in cases involving progression to periodontitis. Meanwhile, the authors of [10] showed that there are no apparent structural changes in the enamel in generalized periodontitis.

An analysis of the literature data showed that, for chronic periodontitis, the changes in hard dental tissues, especially in enamel, are unconfirmed, and having information about the changes in the composition of tooth enamel could allow the development of a noninvasive method to diagnose this disease and provide a correct treatment plan. Therefore, research of the tissues of teeth with periodontitis is urgently needed.

Biochemical analysis, scanning electron microscopy [10], fluorescence [7], and spectroscopy [10] are well-known current methods used for tooth tissue research. Biochemical analysis and scanning electron microscopy (SEM) provide quality images of the tooth tissue microstructure and are the most widely used for assessing tooth structure, but they require destructive preparation of the sample [9,10,12,13]. Fluorescence diagnosis in dentistry is based on the analysis of the spectra of fluorescence of hard dental tissues. While the main studied substance is hydroxyapatite, of which teeth are composed, detailed analysis of the composition of teeth is not possible [7]. The limitations of these hard dental tissue research methods could be overcome with the use of Raman spectroscopy. This is a simple, noninvasive, and rapid way of assessing dental tissue [9,12,13].

In [12], with the use of traditional routine histological methods and Raman spectroscopy, comparative research on mineralized tissues of the human jaw was carried out, and it was shown that the joint use of these methods allows significantly more data about pathological processes in the mineralized tissues (in the case of caries) to be collected, as well as allowing the features of mineralization under the conditions of directed bone regeneration to be defined. The authors of [9] studied the processes of bone healing and regeneration in periodontitis treatment using Raman spectroscopy. In our previous work [13], we used Raman spectroscopy to analyze the structure of teeth compared with synthetic apatites. Spectral lines related to the hard and soft tissues of teeth that provide important data for understanding the chemical structural properties of dentin and enamel were discussed. In [6,14,15,16], attention was paid to the study of the periodontal ligament after the application of orthodontic force and gingival slit fluid in periodontal disease. The authors showed the possibility of using Raman spectroscopy to monitor the periodontal condition at the biochemical level in subjects undergoing orthodontic treatment.

The aim of this work is to study the changes in the tissues of teeth with periodontitis using the Raman spectroscopy method for early, rapid diagnosis and the correction of treatment.

## 2. Materials and Methods

A randomized study design was used. Forty-two teeth (molars, premolars, and canines) from European patients aged 35–70 of both genders, that were removed due to chronic periodontitis (26 teeth) or for orthodontic reasons (control group, 16 teeth), were used as the materials of the study. Diagnosis of periodontitis was done clinically and after cone beam computed tomography (CT) analysis (the code of the disease according to ICD-10 (1997)—K05.3). The teeth removed due to severe chronic periodontitis with periodontal pockets of at least 6 mm deep and pathologic tooth mobility of grades III–IV were selected for the main group of study. Computed tomography showed a decrease in the bone tissue around the roots of removed teeth of more than half of the root length.

The study was carried out in accordance with the Declaration of Helsinki. The protocol was approved by the Ethics Committee (extract 20.05.2020 No. 207 of minutes of the meeting of the Committee on Bioethics of Samara State Medical University). The samples were collected within a period of 2 months. Measurements were taken immediately after the sampling.

The surfaces of teeth in 5 different areas were studied: enamel (a), dentin (b), in longitudinal slices), cementum (c), and dental calculus localized in the outer part of the teeth. The degree of intensity of the surface formation of the studied teeth corresponded to distinct under-gum (e) and above-gum (d) calculus [17].

**Figure 1:**
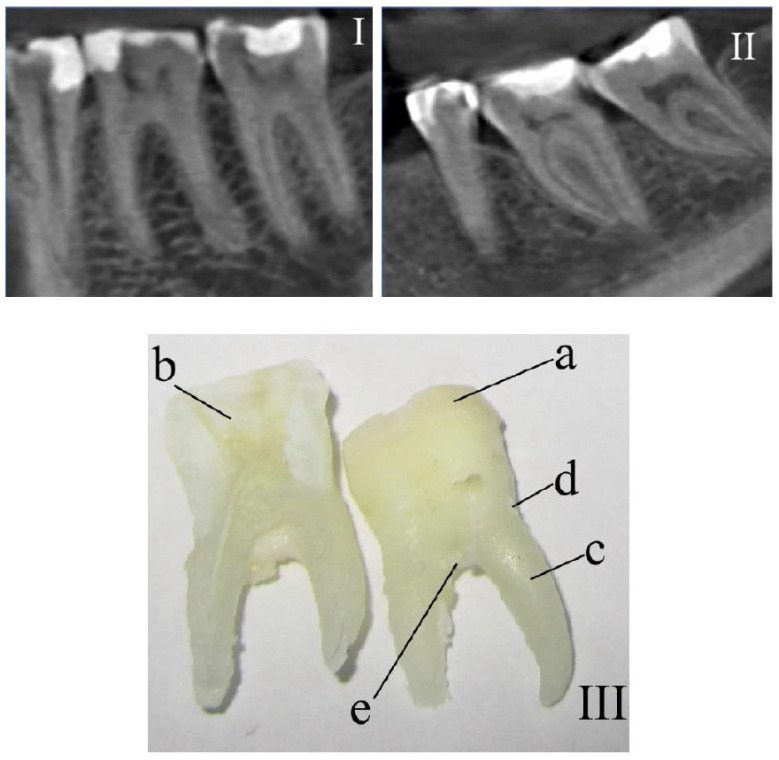
The fragments of teeth following computed tomography that were (**I**) healthy and (**II**) diagnosed with periodontitis. (**III**) Photo of a tooth with the researched areas indicated: a—enamel, b—dentin, c—cementum, d—above-gum dental calculus, and e—under-gum dental calculus.

Three spectra were investigated (with subsequent averaging) in every studied area at 3–5 different points of the surface of every tissue of each tooth. Samples were divided into 2 main groups: the control group (Figure 1I) and the group with periodontitis (Figure 1II).

An in vivo study of the enamel of 22 teeth (molars, premolars, and canines) of one female volunteer patient was also carried out. One of the patient’s teeth was diagnosed with localized periodontitis (disease code according to ICD-10 (1997)—K05.3).

The study was carried out using Raman spectroscopy, implemented using the process described in detail in [18].

The experimental process included the use of a semiconductor laser (LML-785.0RB-04, California, USA), an optical module for Raman spectroscopy (RPB-785, Changchun, China), a spectrograph (Sharmrock SR-303i, www.andor.oxinst.com) with an integrated digital camera (ANDOR DV-420A-OE, www.andor.oxinst.com) that was cooled to −60 °C, and a computer.

The use of this spectrograph provided a wavelength resolution of 0.15 nm with a low level of inherent noise. The method of subtracting the fluorescence component of polynomial approximation with additional filtration of random noise effects was used to exclude autofluorescence from the Raman spectrum. Analysis of the Raman spectra was carried out in the range of 350–2200 cm^−1^ in this work. The power of the laser radiation, 400 mW, within the used exposure time (30 s) did not cause any changes to the samples. The optical probe, positioned over the subject at a distance of 7 mm, was used for Raman spectrum registration [19].

## 3. Results

**Figure 2:**
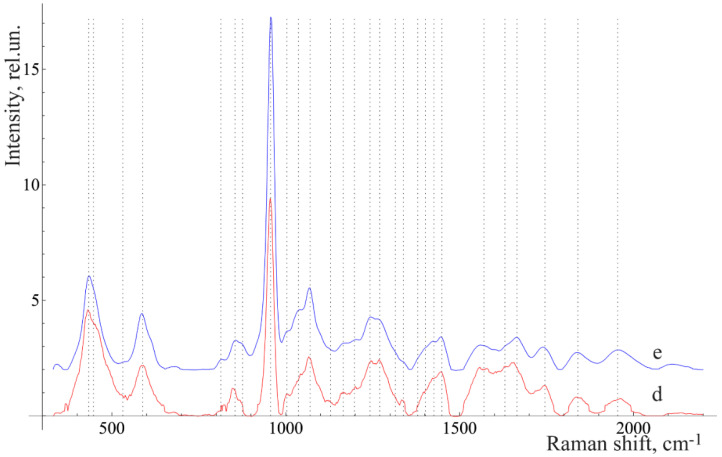
The average Raman spectra, normalized to the average intensity of the studied samples. d—above-gum dental calculus and e—under-gum dental calculus.

We considered the characteristic average normalized Raman spectra of surface formations on teeth with this disease (Figure 2). This is often the reason for this disease.

Figure 2 shows that the Raman spectra of the under-gum and above-gum calculi have certain spectral features that are apparently related to different periods of disease formation. In the initial stage of dental calculus formation, the above-gum calcareous deposits are composed primarily of organic components, as can be seen from the more intense lines in the ranges of 1550–1565 cm^−1^ (Amide II) and 1600–1665 cm^−1^ (Amide I) and the less intense line at 956 cm^−1^ (PO_4_^3−^ (ν_1_), hydroxyapatite), compared with the under-gum calculus spectrum. At the same time, the under-gum calculus spectrum is characterized by the explicit intensity of the lines of mineral components (PO_4_^3−^ (ν_1_), hydroxyapatite).

**Figure 3:**
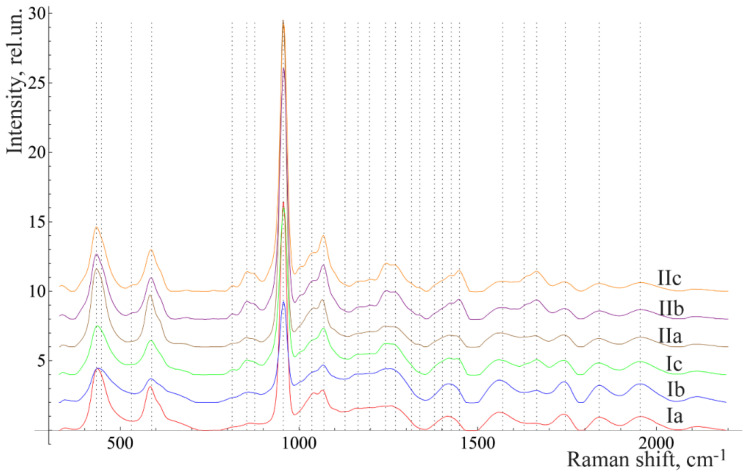
The average Raman spectra, normalized to the average intensity, for two groups of samples studied in vitro: a—enamel, b—denti and c—cementum. I = healthy, while II = diagnosed with periodontitis.

**Figure 4:**
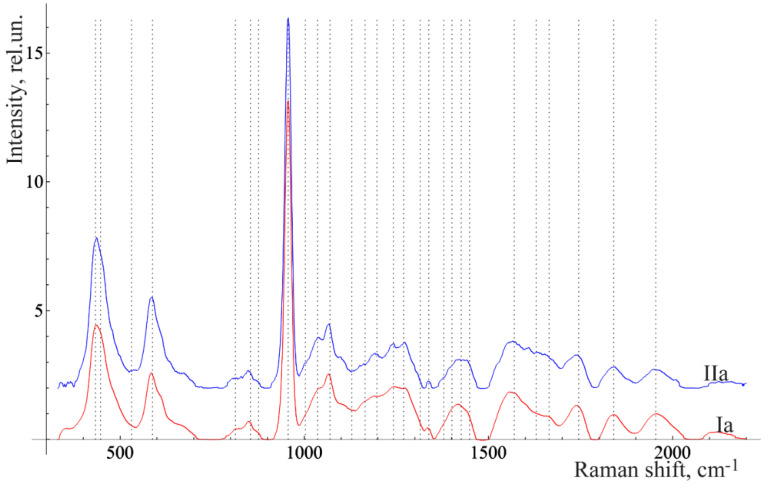
The average Raman spectra, normalized to the average intensity, of two in vivo studied groups of teeth of the volunteer: Ia—healthy enamel and IIa—enamel of the teeth with periodontitis.

Figure 3 and Figure 4 show the averaged spectra of the tissues of teeth with periodontitis and healthy teeth from the in vitro study (Figure 3) and the in vivo study (Figure 4).

The analysis of the healthy tooth tissues and the tissues of teeth with periodontitis showed that the main spectral features of tissues of teeth with periodontitis are changes in the intensity of organic compound lines at 852, 873 cm^−1^ (C–C stretching, proline, and hydroxyproline (collagen assignment)) [20], 1664 (Amide I), 1242 (Amide III) [21], and 1446 cm^−1^ (lipids and proteins) [22], as well as changes in the intensity of the lines of mineral compounds of the teeth at 956 cm^−1^ (P–O symmetrical valence fluctuation PO_4_^3−^ (ν_1_)) [23].

The comparative analysis shown in Figure 2, Figure 3 and Figure 4 highlights many spectral changes in all tissues of teeth with periodontitis. These changes mainly occur in the same Raman lines as those related to calculus.

These spectral features are likely to be related to biochemical processes that take place during the formation of surface deposits during periodontitis (e.g., dental calculus and plaques), which affect all tooth tissues. The etiology of calculus formation is related to the mechanism of mineralization of the tooth surface deposits that consist of hydrocarbons and proteins (30% of each), as well as about 15% of lipids. The other components are extracellular bacterial products (plaques), remnants of their cytoplasm, and cell membranes (extracellular polysaccharides) [17].

**Figure 5:**
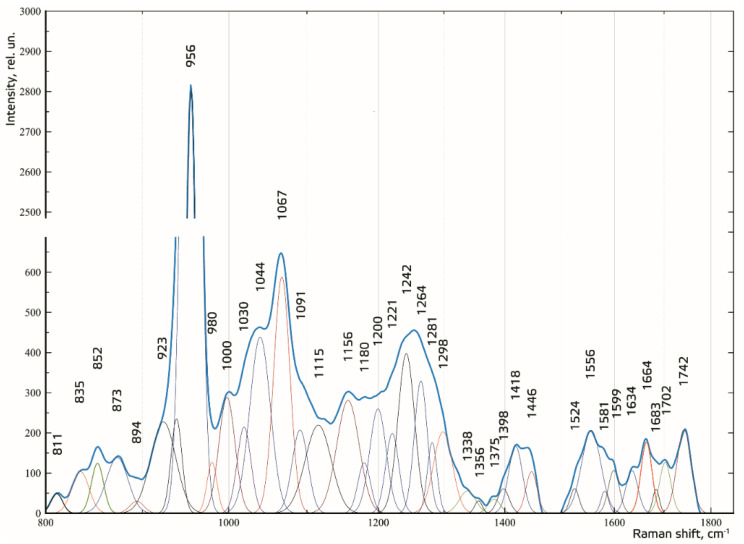
Spectral contour distribution of the enamel samples. The blue line is the original spectrum.

To make the received Raman spectra more informative, a nonlinear regressive analysis of the Raman spectra was conducted, including an investigation of their spectral line decomposition. Figure 5 shows the results of decomposition of the spectral contours on the sum of distribution of the Gaussian lines. The Gaussian test function is described by the formula in [24].

The composition of the spectral lines was determined by literature analysis and multi-iteration modeling of 392 Raman spectra using MagicPlotPro 2.5.1 software. When modeling the spectral contours at the lines used as a template, the position x_0_ and the width of the line (HWHM—half width at half) *dx* were fixed. Only the intensity of the line was selected when modeling. This allowed us to achieve highly stable results when modeling the contours. The amplitude of the lines *a*, which depended on the values of the independent regressors *dx* and *x_0_*, as defined in the initial terms of the analysis, was used as a criterion variable.

The average value of the coefficient of determination for the initial result spectrum in the range of 780–1780 cm^−1^ was R^2^ = 0.998, the relative spectral line intensity assessment error *a* was less than 8%, the average standard deviation of the coordinate of a line *x_0_* was 1.4 cm^−1^, and the average standard deviation of the width of the Gaussian line (HWHM) *dx* was 2.3 cm^−1^.

For the relative quantitative analysis of the component composition, the relative coefficient *k* was introduced, where the Raman line of amide *I* ~1664 cm^−1^ was used as a denominator:

(1) $$k_{i}=\frac{I_{i}}{I_{1664}},$$

where *I_i_* represents the values of intensity of the spectral lines of the analyzed components.

The analysis of the received data was done with IBM SPSS Statistics software through linear discriminant analysis (LDA).

**Figure 6:**
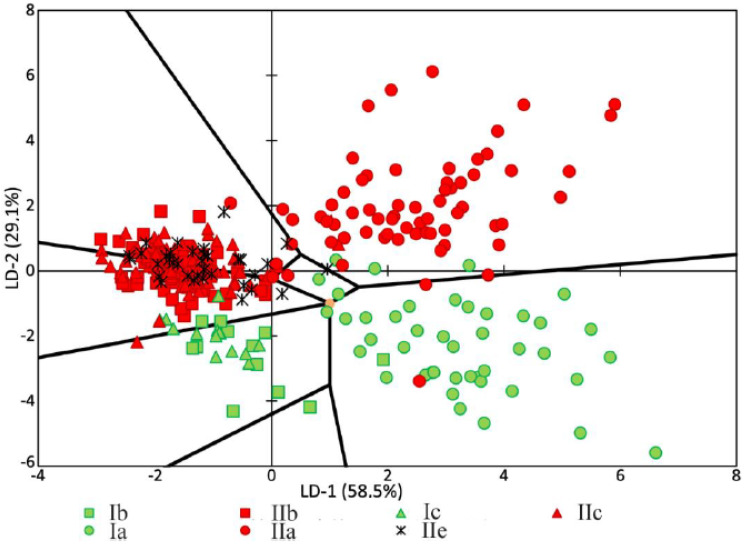
Chart of values showing the linear discriminant functions of the tooth tissue samples. a—enamel, b—dentin, and c—cementum. I = healthy tissue, II = tissue diagnosed with periodontitis.

The analysis of the relationships among groups with a pathology or relation to a certain tooth tissue is shown in Figure 6. It can be seen that most of the dispersion between the studied groups of samples can be described by the LD-1 function (58.5%). The common sampling size was 392 Raman spectra. The discriminant function LD-2 was able to describe 29.1% of the dispersion. This function has the physical meaning of the relationship of tooth tissue to the healthy group or to the group with periodontitis.

Positive values of LD-1 were found to mainly characterize the Raman spectra received from the enamel samples, and vice versa; the negative values characterized the samples of cementum, dentine, and dental calculus. The areas of the groups showed intersections, which influenced the rate of correctly classified subjects. The LD-1 function has the physical meaning of the difference between spectral compositions of tooth tissues. Positive values of LD-2 characterized the Raman spectra of the tooth tissue with periodontitis, and the negative values characterized the Raman spectra of healthy tooth tissue.

**Figure 7:**
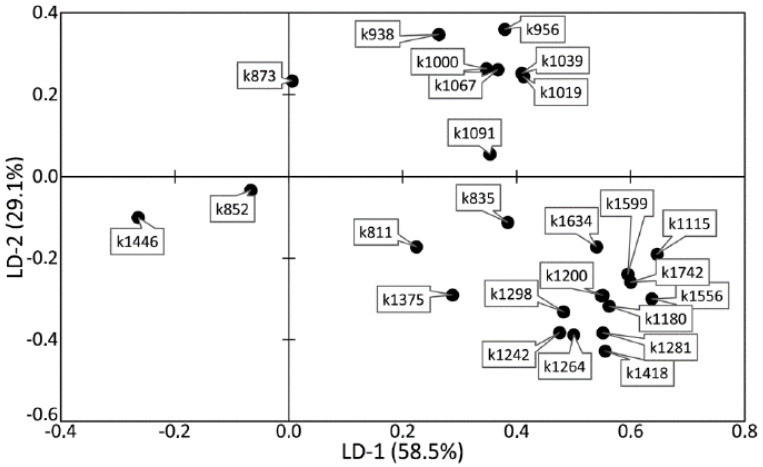
The values of factor structure coefficients for the tooth tissue samples.

Figure 6 and Figure 7 show that the difference between healthy tissues and tissues with periodontitis can be described by the LD-2 function. It can be noted that the spectral composition of dental calculus showed similar changes to the spectra of dentin and cementum, which confirms the earlier hypothesis that calculus influences the internal structures of tooth tissue.

High relative intensity values were observed for the lines ~1446 (CH_2_ scissoring and CH_3_ bending fluctuations of lipids and proteins), ~852 (C–C stretching benzene ring of proline), and ~873 cm^−1^ (C–C stretching benzene ring of hydroxyproline), with the rest of the lines having low spectral lines. These values characterize the tooth tissues—dentin, cementum with periodontitis, as well as calculus—compared with enamel, which indicates the differences in the organic–mineral compositions of these tissues.

**Figure 8:**
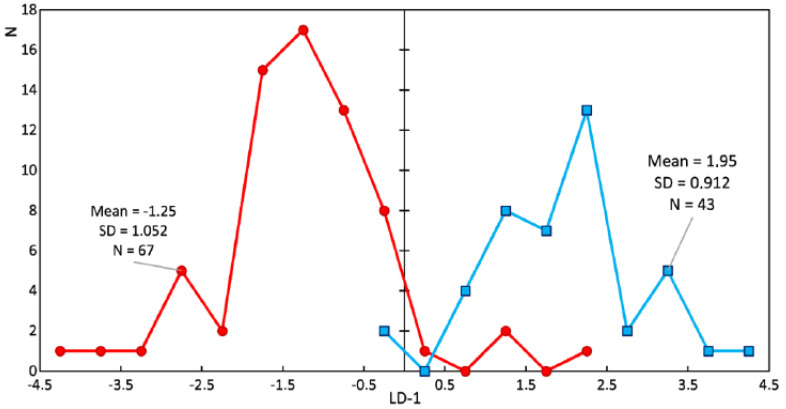
Chart of the linear discriminant function values of the enamel samples. The red line is the enamel of teeth with periodontitis (damaged enamel), and the blue line is the enamel of healthy teeth.

**Figure 9:**
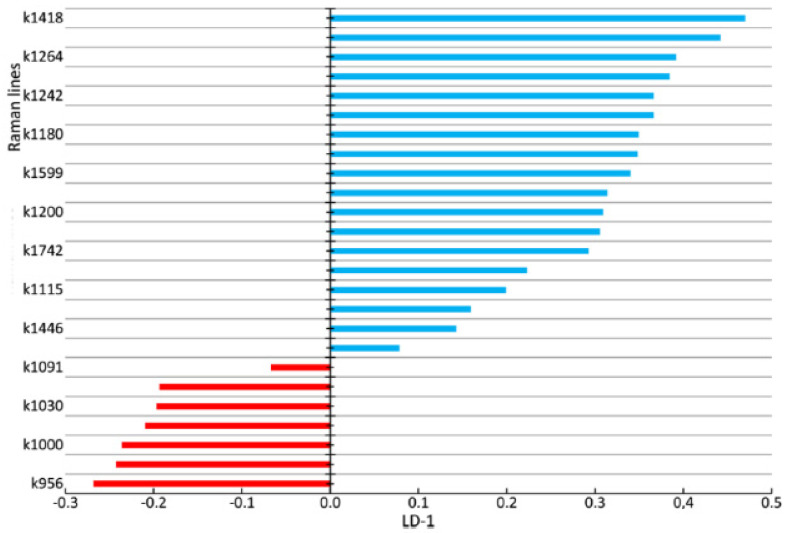
The values of factor structure coefficients for the enamel samples. Negative values are highlighted in red and positive values are highlighted in blue.

Study of the changes in the enamel of teeth with periodontitis was further carried out. Figure 8 and Figure 9 show a comparison of the LDA results of the enamel of healthy teeth and teeth with periodontitis. Sixty-seven spectra of the enamel of teeth with periodontitis and 43 Raman spectra of the enamel of healthy teeth were analyzed. The discriminant function LD-1 was able to describe 100% of the dispersion. Positive LD-1 values characterized the Raman spectra of the healthy enamel samples (the average LD-1 value of the group was 1.95, and the standard deviation was 0.912), and vice versa; negative values characterized the Raman spectra of the group of pathologic enamel samples (the average LD-1 value of the group was −1.25, and the standard deviation was 1.052). The areas of the groups had a minor intersection in the range of LD-1 = (−0.25; 2.25).

Figure 9 shows the coefficients of the factor structure matrix, with a correlation between the variables in the model and the discriminant function. In the analysis, these correlation coefficients were considered to be the factor loadings of the variables for each discriminant function.

The higher the absolute value of LD-1 for the variable is, the more strongly it determined the difference between the groups of samples in the received model of discriminant analysis. For example, the values of the introduced coefficients k873, k956, k1000, k1039, k1044, k1067, and k1091 were higher in the group of enamel samples with periodontitis, which indicates an increase in the relative intensity of the corresponding lines in tissue with periodontitis.

The increase in the relative intensity of the lines for hydroxyapatite 956 (P–O symmetrical valence fluctuation PO_4_^3−^ (ν_1_)), ~1044 (PO_4_^3−^ (ν_3_) (P–O asymmetrical valence fluctuation)), 1067 (C–O planar valence fluctuation CO_3_^2−^ (ν_1_) B-type substitution), and 1091 cm^−1^ (C–O planar valence fluctuation CO_3_^2−^ (ν_1_) A-type substitution) may be related to the presence of a water–mineral metabolism disorder in the tissues of teeth with periodontitis, which leads to more intensive substitution of the hydroxide ion OH by apatite ions CO_3_^2−^ in the structure.

The change in the relative intensity of the lines at 1000 cm^−1^ and 1039 cm^−1^, corresponding to fluctuations in the phenylalanine molecule, and 873 cm^−1^ (C–C stretching, proline and hydroxyproline (collagen assignment)) are apparently related to collagen synthesis disorder, which can also be seen in osteoporotic changes of bone tissues, as we showed earlier in [25].

We also observed a reduction in the relative intensity of the lines at ~1742 (phospholipids), ~1556 (Amide II Parallel/Antiparallel β-sheet structure), 1200–1300 (Amide III), ~1418, and ~1446 cm^−1^ (CH_2_ scissoring and CH_3_ bending fluctuations of lipids and proteins) in the tissues of teeth with periodontitis compared with healthy tissues. This effect may have been caused by the dehydration of peptide groups of amides that are sensitive to structural changes in the molecules of collagen [26].

In [6,16], chemical and structural changes were shown in the periodontal ligament after the application of orthodontic force and gingival slit fluid in teeth with periodontitis. Violation of the ligamentous apparatus leads to the development of periodontitis and changes in tooth tissues. Raman spectroscopy analysis of enamel can be used for the early diagnosis of periodontitis.

As a result of the discriminant analysis, we built a discriminant model of the enamel of healthy teeth and the enamel of teeth with periodontitis, taking into account characteristic changes in the relative intensity of the Raman lines. The number of true positive (*TP*) results was 64, while there were 3 false negative (*FN*) results. The number of true negative (*TN*) results was 41, and there were 2 false positive (*FP*) results.

The calculated sensitivity and specificity values of the method are

(2) $$Sen=\frac{TP}{TP+FN}=\frac{64}{64+3}=95.5\%$$

(3) $$Spe=\frac{TN}{TN+FP}=\frac{41}{41+2}=95.3\%.$$

**Figure 10:**
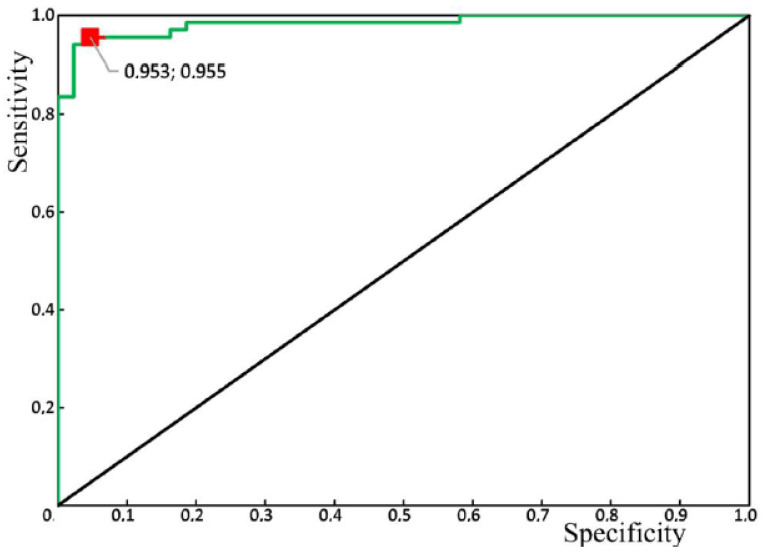
Receiver operator characteristic (ROC) analysis of the algorithm for periodontitis assessment, using the Raman spectroscopy method: green line—ROC-curve, the red square is the optimal cut-off point.

**Figure 11:**
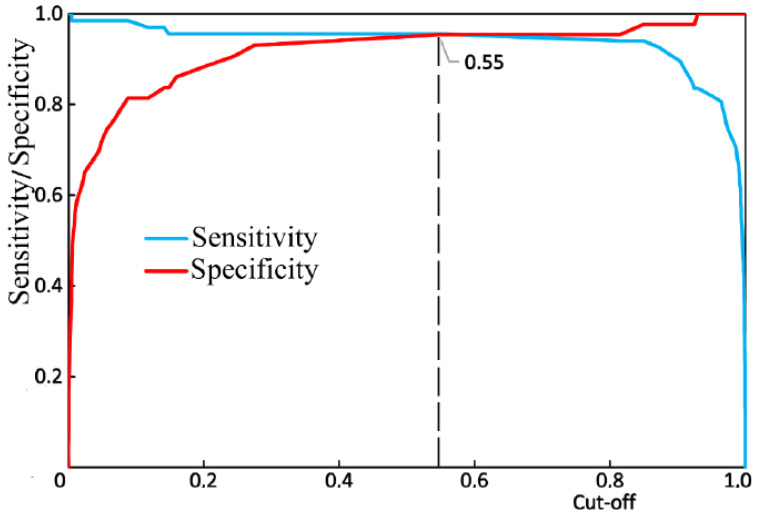
The balance point between sensitivity and specificity.

Figure 10 shows the results of the receiver operator characteristic (ROC) analysis of the developed algorithm for diagnosing periodontitis. The discriminant adequacy of the method had an area under the curve (AUC) value of 0.983, which indicates the great quality of the diagnostic tool. The standard error (SE) was 0.01, and the 95% confidence interval of the AUC was in the range of 0.963–1. The optimal cut-off point for the presented algorithm, determined according to the condition of balance between sensitivity and specificity, was 0.55 (Figure 11). The values of sensitivity and specificity for the diagnostic model at that cut-off point were 95.5% and 95.3%, respectively.

Therefore, if the received spectrum of enamel is classified as the spectrum of enamel with periodontitis, it could be a reason for including the patient in the at-risk group and may determine the treatment given.

## 4. Discussion

The main spectral changes in the tissues of teeth with periodontitis were identified in this work. The etiology of these changes is connected to the formation of calculus on the surface of teeth which, in turn, results in structural changes to all tooth tissues with periodontitis. These changes occur due to the presence of a water–mineral metabolism disorder in the tooth tissues (intensive substitution of hydroxide ion OH by apatite ions CO_3_^2−^ in the structure) or collagen synthesis disorder. Similar changes occur in bone tissues with osteoporosis, as we presented earlier in [25].

The spectral changes found in this work that occur in periodontitis do not occur in other widespread dental diseases (e.g., caries). We previously carried out studies [27] that showed a decrease in the concentration of ions (PO_4_)^3−^ in caries, as also shown in [28].

Diagnosing the spectral changes in tooth enamel, as well as developing an algorithm to identify enamel with periodontitis, will allow at-risk patients to be identified and treated with hydroxyapatite. The sensitivity and specificity values of the developed algorithm were 95.5% and 95.3%, respectively.

The received results are a prerequisite for creating an express device for the noninvasive (in vivo) assessment of periodontitis, based on changes in tooth enamel spectral values. These studies have already been carried out in vivo in this work and have shown good results, similar to the results of in vitro studies.
